# Persistent Chronic Thrombo-Inflammation in Anti–Glomerular Basement Membrane Disease Despite Immune Complex Removal

**DOI:** 10.1016/j.ekir.2025.06.014

**Published:** 2025-06-19

**Authors:** Mélodie Douté, Linnéa Tyrberg, Younès Youssfi, Ingeborg Bajema, Johan Mölne, Antonino Nicoletti, Giuseppina Caligiuri, Mårten Segelmark, Marc Clement

**Affiliations:** 1 Université Paris Cité and Université Sorbonne Paris Nord, INSERM, LVTS, F-75018 Paris, France; 2 Laboratoire d'Excellence INFLAMEX, Paris, France; 3 Department of Clinical Sciences Lund, Lund University, Lund, Sweden; 4 Department of Specialized Medicine, Helsingborg Hospital, Helsingborg, Sweden; 5 Center for Research in Economics and Statistics, National School for Statistics and Economic Administration, Polytechnic Institute of Paris, Palaiseau, France; 6 Department of Pathology and Medical Biology, University of Groningen, University Medical Center Groningen, The Netherlands; 7 Department of Clinical Pathology, Sahlgrenska University Hospital, Region Västra Götaland, Gothenburg, Sweden; 8 Department of Cardiology, Assistance Publique-Hôpitaux de Paris, Hôpitaux Universitaires Paris Nord Val-de-Seine, Site Bichat, Paris, France; 9 Department of Nephrology, Skåne University Hospital, Lund, Sweden

**Keywords:** anti-GBM disease, hematopoietic growth factors, imlifidase, platelet activation, thrombo-inflammation

## Abstract

**Introduction:**

Anti–glomerular basement membrane (anti-GBM) disease is a severe autoimmune disease characterized by autoantibody-mediated glomerular damage, leading to a rapid decline in kidney function and end-stage renal disease. The stimulation of megakaryopoiesis (Mkpoiesis) and platelet production, driven by kidney-derived hematopoietic growth factors (HGFs) such as thrombopoietin (TPO), exacerbates chronic thrombosis and inflammation. Activated platelets release bioactive molecules able to promote microvascular dysfunction, cell proliferation, and excessive extracellular matrix deposition in injured glomeruli. Understanding the molecular mechanisms driving persistent thrombo-inflammation during anti-GBM disease will enhance therapeutic improvements. The hypothesis of this study was that anti-GBM disease could stimulate the production of kidney-derived HGFs and proinflammatory mediators. In addition, immobilized anti-GBM antibodies in glomeruli could directly activate FcγRIIA expressing platelets, thereby promoting chronic platelet activation during anti-GBM disease.

**Methods:**

In the GOOD-IDES-01 trial, patients received, in addition to standard care, imlifidase (the IgG-degrading enzyme, IdeS). In plasma samples collected from patients with anti-GBM disease, before and after imlifidase treatment, and from healthy blood donors (HBDs), we analyzed plasma HGFs, proinflammatory and platelet activation markers, and platelet-derived products.

**Results:**

Anti-GBM disease significantly elevated plasma proinflammatory and platelet activation markers, and HGFs (TPO and stem cell factor [SCF]). Plasma TPO correlated with anti-GBM titers. Standard care and imlifidase treatment only reduced TPO levels and platelet counts. Platelet activation markers (CD62P and Tlt1) strongly correlated with platelet-derived products (PDGF, CCL5, PF4, and TGFβ) during the active phase of the disease, but remained elevated despite the treatment.

**Conclusion:**

Circulating HGFs, proinflammatory cytokines, and platelet activation markers are important biomarkers of anti-GBM disease activity. Chronic platelet activation, persists independently of anti-GBM antibody integrity, highlighting the need for therapies targeting thrombo-inflammation.

## Graphical abstract

**Figure:**
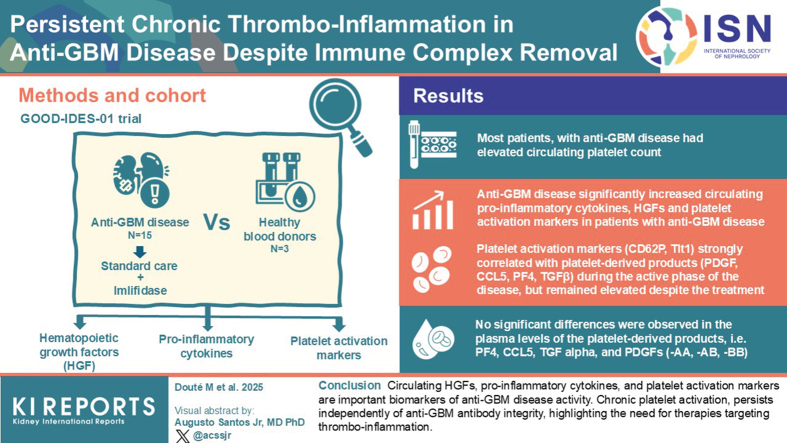

Anti- GBM disease is characterized by the presence of autoantibodies directed against the noncollagenous domain of the α3-chain of collagen IV.^1^, ^2^, ^3^ Their fixation in glomeruli activates the complement system and recruits immune cells, instigating inflammation. This inflammatory response triggers the formation of cellular crescents, culminating in swift and severe kidney damage. Patients typically exhibit symptoms of acute renal failure, and occasionally, pulmonary hemorrhage. Despite the implementation of treatments such as corticosteroids, immunosuppression, and plasma exchange, the rate of renal survival remains at a mere 26% to 30%, depending on the renal function at the time of diagnosis.^2^^,^^4^^,^^5^ Current treatments seem to be inefficient in preventing autoantibodies deposition or removing them from the GBM to ameliorate disease evolution. A promising therapeutic strategy involves the use of imlifidase, a drug based on the bacterial enzyme IdeS, with the ability to selectively cleave circulating and tissue-bound IgG antibodies, within a few minutes following administration.^6^, ^7^, ^8^ The GOOD-IDES-01 trial examined the safety and efficacy of imlifidase, yielding encouraging results; 67% of the patients regained enough renal function to no longer require dialysis.^9^^,^^10^ Despite these positive outcomes, patients in the GOOD-IDES-01 trial still experienced some degree of chronic renal damage. This suggests that elements beyond the swift removal of autoantibodies could play a role in the progression of the disease.

Among the underappreciated factors that could contribute to glomerular inflammation, and pathological tissue remodeling in the pathophysiology of anti-GBM disease, platelets emerge as an element of interest to consider. Platelets, beyond their crucial role in hemostasis, can be activated by multiple pathways, and are increasingly recognized as crucial players in autoimmunity, allo-immunity, cancer, and immunity against pathogens.^11^^,^^12^ Importantly, platelets can interact with IgGs bound to their antigens, through their FcγRIIA.^13^

Upon interaction with antibody-antigen complexes, the FcγRIIA from platelets triggers the activation of the Syk-PLCg2 signaling pathway resulting in intracellular calcium mobilization, a-granule secretion and activation of the integrin GpIIbIIIa, allowing platelets to bind fibrinogen, and form thrombi.^14^ Soluble immune complexes stimulate platelets and contribute to the pathogenesis of anaphylaxis, heparin-induced thrombocytopenia, and lupus nephritis.^15^, ^16^, ^17^, ^18^ However, whether autoantibodies bound to the GBM trigger platelet activation during anti-GBM disease in human remains to be established.

The potent role of platelets in anti-GBM disease is supported by the biological activity of the cargo of molecules they release following activation. Upon a-granule secretion, CD62P and Tlt1 are exposed at their surface, and released in the extracellular space making them potential biomarkers of systemic platelet activation. Both molecules stimulate platelet interaction with leukocytes, and promote their recruitment to sites of inflammation.^19^, ^20^, ^21^, ^22^, ^23^ CD62P promotes glomerular inflammation in experimental anti-GBM disease^24^; the function of Tlt1 has not yet been investigated in this pathology. In addition, a-granule secretion releases potent bioactive molecules, including the promitotic platelet-derived growth factors (PDGFs); the monocyte and T cell chemoattractant, CCL5; the profibrotic, TGFβ; and the proinflammatory and prothrombotic, platelet factor 4 (PF4). We recently showed that chronic platelet activation in experimental anti-GBM disease promotes chronic glomerular thrombo-inflammation and renal fibrosis.^25^^,^^26^

Chronic platelet activation consumes platelets; thus, their production needs to be boosted to keep a minimal level of circulating platelets and avoid spontaneous bleeding. Platelets are generated by megakaryocytes. Mature, platelet-generating megakaryocytes, are produced in hematopoietic tissues through MKpoiesis.^27^ HGFs such as TPO and SCF, among others, regulate MKpoiesis and platelet production.^28^^,^^29^ The liver and kidneys are the primary producers of TPO under steady-state conditions. SCF is primarily produced in the bone marrow by endothelial cells. Under proinflammatory conditions, MKpoiesis and platelet production can be enhanced. For example, IL1α can trigger a TPO-independent burst of megakaryocytes in the bone marrow, whereas IL6 enhances the secretion of TPO by hepatocytes.^30^^,^^31^

Platelets contribute to the acute glomerular injury in anti-GBM models.^24^^,^^32^^,^^33^ Recently, we discovered that platelets and MKpoiesis drive chronic glomerular lesions and pathological tissue remodeling during anti-GBM disease. We showed that the inflamed kidney is a major source of TPO during experimental anti-GBM disease, as well as in patients with anti-neutrophil cytoplasmic autoantibody vasculitis–associated glomerulonephritis.^25^ As a result, MKpoiesis and platelet production were boosted, resulting in thrombocytosis. In both patients and experimental anti-GBM disease, platelets continuously accumulated in injured glomeruli. This process contributed to chronic platelet activation, alongside microvascular inflammation, renal dysfunction, and TGFβ-induced glomerular remodeling in experimental anti-GBM disease, demonstrating that chronic thrombo-inflammation is a hallmark of anti-GBM disease. TPO neutralization efficiently prevented this vicious circle. Therefore, excessive Mkpoiesis and platelet production and activation, stimulate kidney disease progression in anti-GBM disease.^25^^,^^26^ Therefore, our study suggested that counteracting HGF-enhanced MKpoiesis and chronic platelet activation are potential therapeutic strategies to curb chronic microvascular thrombo-inflammation in anti-GBM disease. Furthermore, markers of platelet activation and HGFs could serve as biomarkers to monitor disease activity and gauge the response to treatments in patients with anti-GBM disease.

These findings raised a new question about the molecular trigger of chronic platelet activation during anti-GBM disease. Given that platelets continuously accumulate in glomeruli and that anti-GBM autoantibodies could activate platelets via the FcγRIIA, we hypothesize that this interaction could promote chronic glomerular thrombo-inflammation. Removing anti-GBM autoantibodies from glomeruli should quickly reduce platelet activation, as well as inflammation. Conversely, if removing autoantibodies does not reduce features of thrombo-inflammation, it would suggest that platelets are activated independently of their presence in patients with established anti-GBM disease.

We tested this hypothesis in the context of the GOOD-IDES-01 clinical trial in patients with anti-GBM disease wherein the benefit of removing all IgGs, including glomerular anti-GBM autoantibodies, was evaluated using imlifidase.

## Methods

### Description of the Cohort

**Figure 1:**
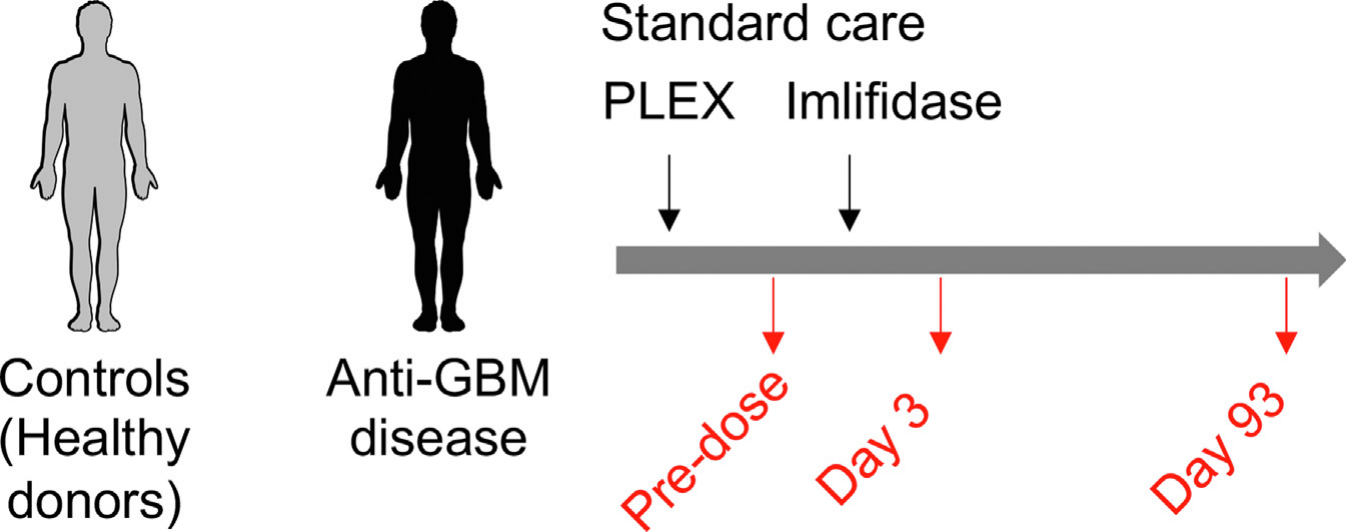
Blood drawing schedule of the GOOD-IDES-01 trial. Anti-GBM disease, anti–glomerular basement membrane disease; PLEX, plasma exchange.

The GOOD-IDES-01 trial (ClinicalTrials.gov: NCT03157037) was previously described.^9^ Briefly, patients with circulating anti-GBM antibodies, and an estimated glomerular filtration rate < 15 ml/min per 1.73 m^2^ were included and treated with a single dose of imlifidase in addition to standard therapy according to local guidelines. Plasma samples from patients (*n* = 15) were purified from blood drawn on ethylenediamine tetraacetic acid and frozen, prospectively for 6 months. In this study, we analyzed samples at the following 3 key time points: before treatment with imlifidase (predose), at day 3 and at day 93 posttreatment (Figure 1). This enabled us to evaluate the changes in the active phase of the disease, and the rapid and long-term effect of the treatment on HGFs, proinflammatory cytokines, and platelet biology, respectively. Control plasma samples were purified from samples of HBDs (*n* = 3) drawn on ethylenediamine tetraacetic acid, and frozen. Study drugs, treatment regimen, follow-up, laboratory analysis, diagnosis, primary and secondary outcomes, and treatment efficiency validation were all previously described.^9^

To prevent any delay before beginning the treatment, kidney biopsies before inclusion in the trial was not a requirement. Therefore, the timing of the kidney biopsies does not match the timing of blood sampling and varies between the patients included in the trial.

Histological analysis, biological parameter acquisition, titration of plasma soluble analytes (cytokines, HGFs, and platelet-related markers) and statistical analysis are described in Supplementary Methods.

## Results

### Patients With Anti-GBM Disease Develop Thrombocytosis

**Figure 2:**
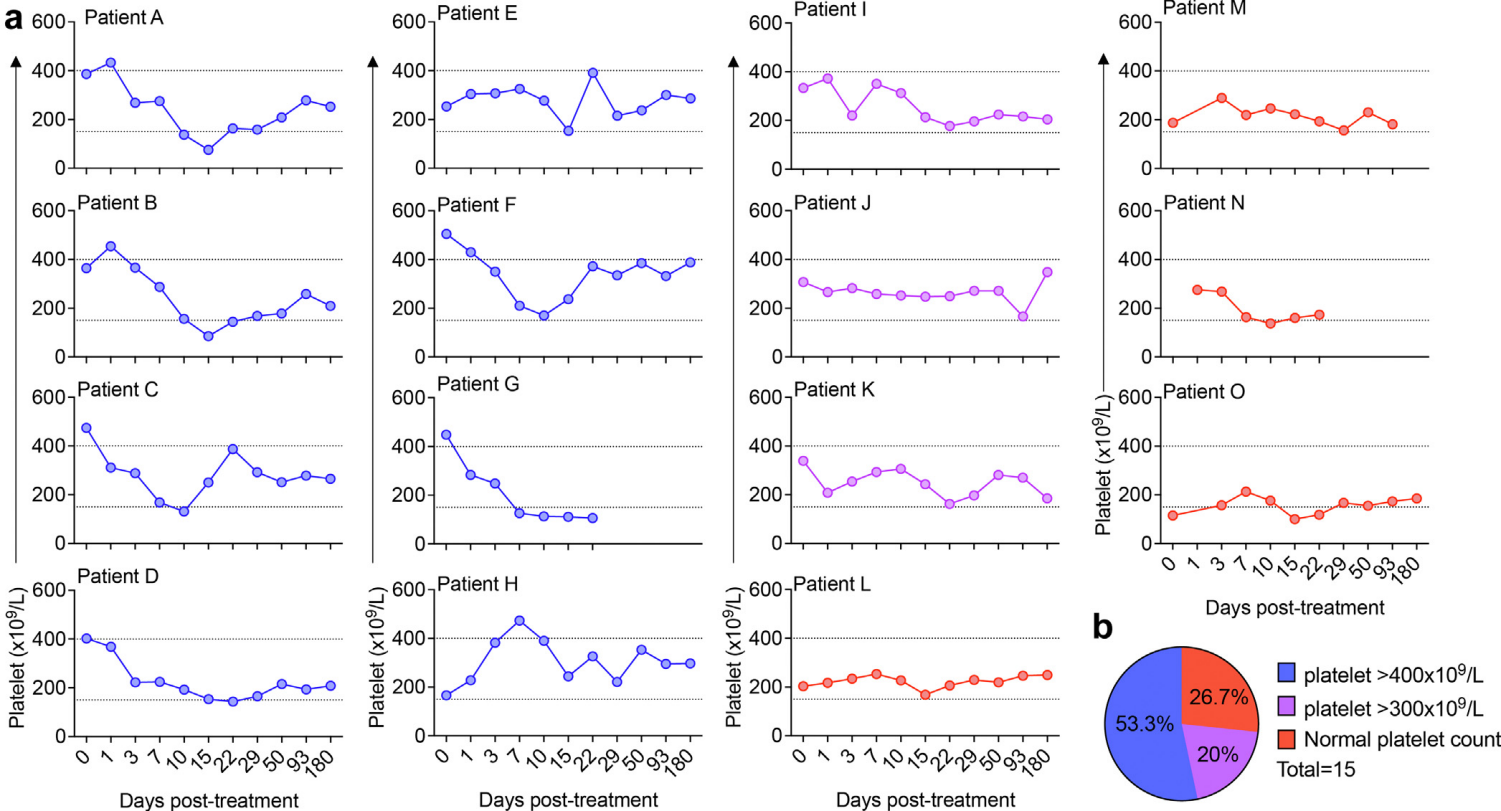
Thrombocytosis is a hallmark of anti-GBM disease. (a) Individual circulating platelet counts over the course of the GOOD-IDES-01 trial. Dotted lines represent normal range (150–400 × 10^9^/l). (b) Distribution of patients reaching high (> 400 × 10^9^/l, blue) and elevated (> 300 × 10^9^/l, purple) platelet counts, and remaining within normal range (> 150 × 10^9^/l, red). Anti-GBM disease, anti–glomerular basement membrane disease.

The analysis of circulating platelet counts revealed that most patients, with anti-GBM disease enrolled in the GOOD-Ides01 study had elevated circulating platelet count (53.3% with platelets > 400 × 10^9^/l, 20% with platelet count 400–300 × 10^9^/l), at some point, during the study (Figure 2a and b). This observation is in line with the observation made in our experimental model of anti-GBM disease.^25^

### Increased Circulating Proinflammatory Cytokines, HGFs, and Platelet Activation Markers in Patients With Anti-GBM Disease

**Figure 3:**
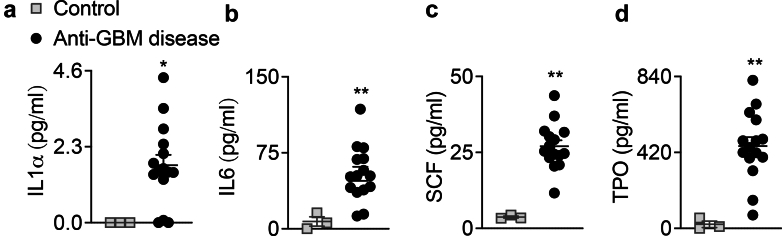
Plasma proinflammatory cytokines and hematopoietic growth factors are increased in patients with anti-GBM disease. Proinflammatory cytokines (a): IL1α, (b): IL6 and hematopoietic growth factors (c): SCF, (d): TPO were titrated in the plasma (ethylenediamine tetraacetic acid) from healthy blood donors (controls, *n* = 3) and patients with anti-GBM disease before pharmacological treatments (*n* = 15). Mann-Whitney test; ∗*P* < 0.05, ∗∗*P* < 0.01. Anti-GBM disease, anti–glomerular basement membrane disease; IL, interleukin; SCF, stem cell factor; TPO, thrombopoietin.

**Figure 4:**
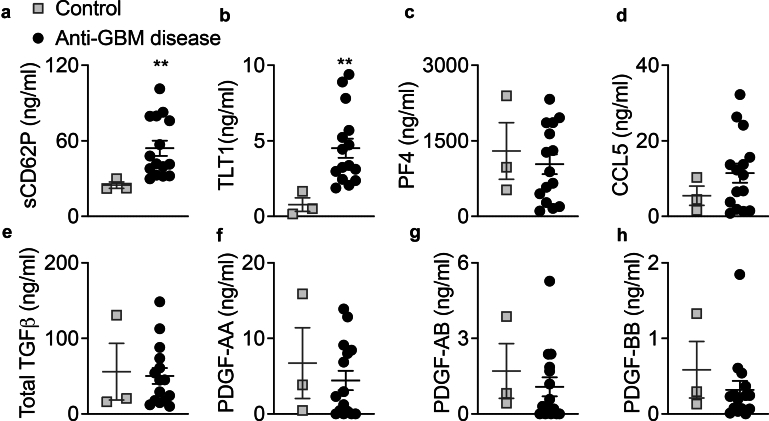
Plasma platelet activation markers are increased in patients with anti-GBM disease. Platelet activation markers (a): sCD62P, (b): TLT1; and platelet-derived products (c): PF4, (d): CCL5, (e): total TGFβ1, (f): PDGF-AA, (g): PDGF-AB, and (h): PDGF-BB were titrated in the plasma (ethylenediamine tetraacetic acid) from healthy blood donors (controls, *n* = 3) and patients with anti-GBM disease before pharmacological treatments (*n* = 15). Mann-Whitney test; ∗∗*P* < 0.01. Anti-GBM disease, anti–glomerular basement membrane disease; CCL5, C-C motif ligand 5; PDGF, platelet-derived growth factor; PF4, platelet factor 4, sCD62P, soluble CD62P; TGFβ1, transforming growth factor beta 1; TLT1, TREM-like transcript 1.

Levels of plasma proinflammatory cytokines, HGFs, platelet activation markers, and platelet-derived products were analyzed in patients and HBDs. Patients with anti-GBM disease displayed significantly higher levels of plasma IL1α, IL6, SCF, and TPO than HBDs (Figure 3a–d). In addition, patients with anti-GBM disease displayed a significant increase in plasma levels of platelet activation markers, that is, sCD62P and TLT1, compared with HBDs (Figure 4a and b). However, no significant differences were observed in the plasma levels of the platelet-derived products,that is, PF4, CCL5, TGFβ, and PDGFs (-AA, -AB, -BB), between both groups (Figure 4c–h). Thus, platelet-derived products are less sensitive than soluble CD62P and Tlt1, to clearly demonstrate a high level of platelet activation.

This first set of data strongly suggests that inflammation, HGFs involved in MKpoiesis, and platelet activation are concomitantly increased in patients with anti-GBM disease.

### Correlative Analysis of Clinical Parameters With Proinflammatory Cytokines, HGFs, and Platelet-Derived Products in Patients With Anti-GBM Disease

To have a better insight into the pathophysiological mechanisms at play during anti-GBM disease, we performed a multivariate analysis of the proinflammatory cytokines, HGFs, and platelet-derived products with the histological (% of cellular, fibrocellular and sclerotic crescent, and of interstitial fibrosis and tubular atrophy) and biological parameters from patients with anti-GBM disease, before imlifidase treatment.

**Figure 5:**
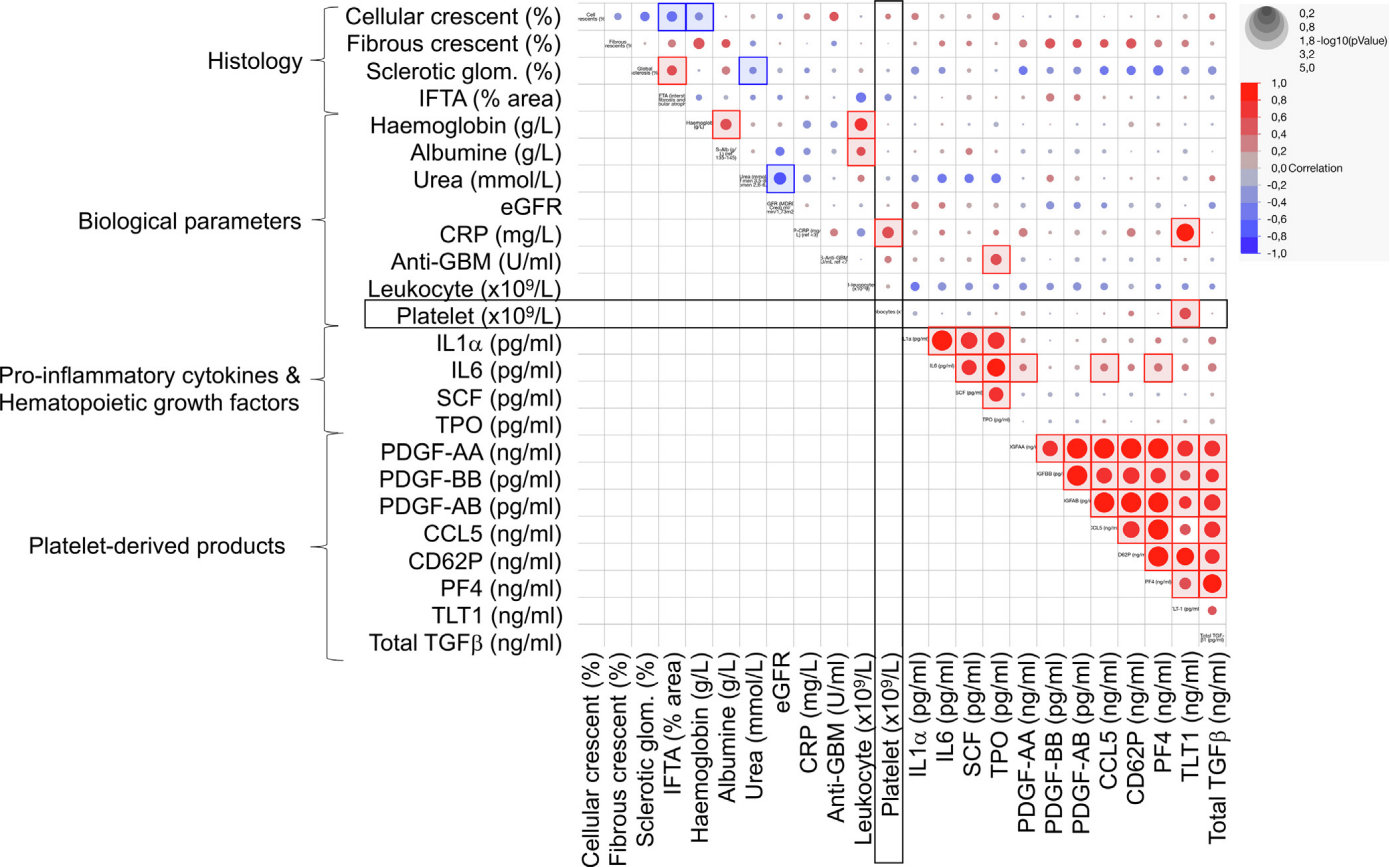
Correlation matrix of clinical and biological parameters in patients with anti-GBM disease. The intensity if the color reflects the coefficient of correlation (Spearman) between variables (0 to +1, red; −1 to 0, blue). Circle size corresponds to the *P* value. Significant correlations are squared (blue for negative correlations, and red for positive correlations). Data displayed are from patients with anti-GBM disease (*n* = 15) in the GOOD-IDES-01 trial before treatment with Imlifidase. Raw data for the multivariate analysis are available in the Supplementary Table S1. Anti-GBM disease, anti–glomerular basement membrane disease; CCL5, C-C motif ligand 5; CRP, c-reactive protein; eGFR, estimated glomerular filtration rate; IFTA, interstitial fibrosis and tubular atrophy; IL, interleukin; PDGF, platelet-derived growth factor; PF4, platelet factor 4; SCF, stem cell factor; TGFβ1, transforming growth factor beta 1; TLT1, TREM-like transcript 1; TPO, thrombopoietin.

Among the clusters of significant correlations that were identified, the most striking one was the highly significant positive correlation (*P* < 0.0001; Spearman coefficient > 0.8) between all the platelet-activation markers, sCD62P and TLT1; and the platelet-derived products PDGFs, CCL5, PF4, and TGFβ1 (Figure 5, Supplementary Table S1). However, these markers were not correlated to histological or biological parameters. This might be because of the heterogeneity of the disease in patients, the natural history of the disease before inclusion in the clinical trial, distinct and shifted kinetics for each process, or the timing of the analysis of the biopsies (before or after blood sampling). In addition, most platelet-derived products (sCD62P, PDGFs, PF4, CCL5, and TGFβ) were not correlated with platelet count, except for TLT1 (*P* = 0.0172, Spearman coefficient = 0.6036), demonstrating that the quantity of circulating platelets is poorly related to their activation status.

Interestingly, platelet count and plasma TLT1 were significantly and positively correlated with plasma c-reactive protein, suggesting that systemic inflammation may contribute to platelet production and activation.

Another highly significant cluster of positive correlations was observed between HGFs (TPO and SCF) and proinflammatory cytokines (IL1α and IL6). This observation strongly suggests that HGFs are induced by proinflammatory signals. Importantly, plasma TPO positively correlated with the level of circulating anti-GBM autoantibodies, suggesting that the intensity of the anti-GBM–induced glomerular injury could be linked to TPO production, as observed in experimental anti-GBM disease.^25^ As observed with the platelet-derived products, platelet counts were not correlated with HGFs or proinflammatory cytokines, possibly because of the heterogeneity in disease intensity, the natural history of the disease before inclusion in the clinical trial, or distinct and shifted kinetics for each process.

### Follow-Up Analysis of Clinical Data in Patients From GOOD-IDES-01: Platelet Count Improves With Immunosuppressive Treatments

Next, we investigated whether the treatment of patients with imlifidase and immunosuppression affected biological parameters, inflammation, and platelet biology. To this end, only patients with paired samples were analyzed (*n* = 12–13).

**Figure 6:**
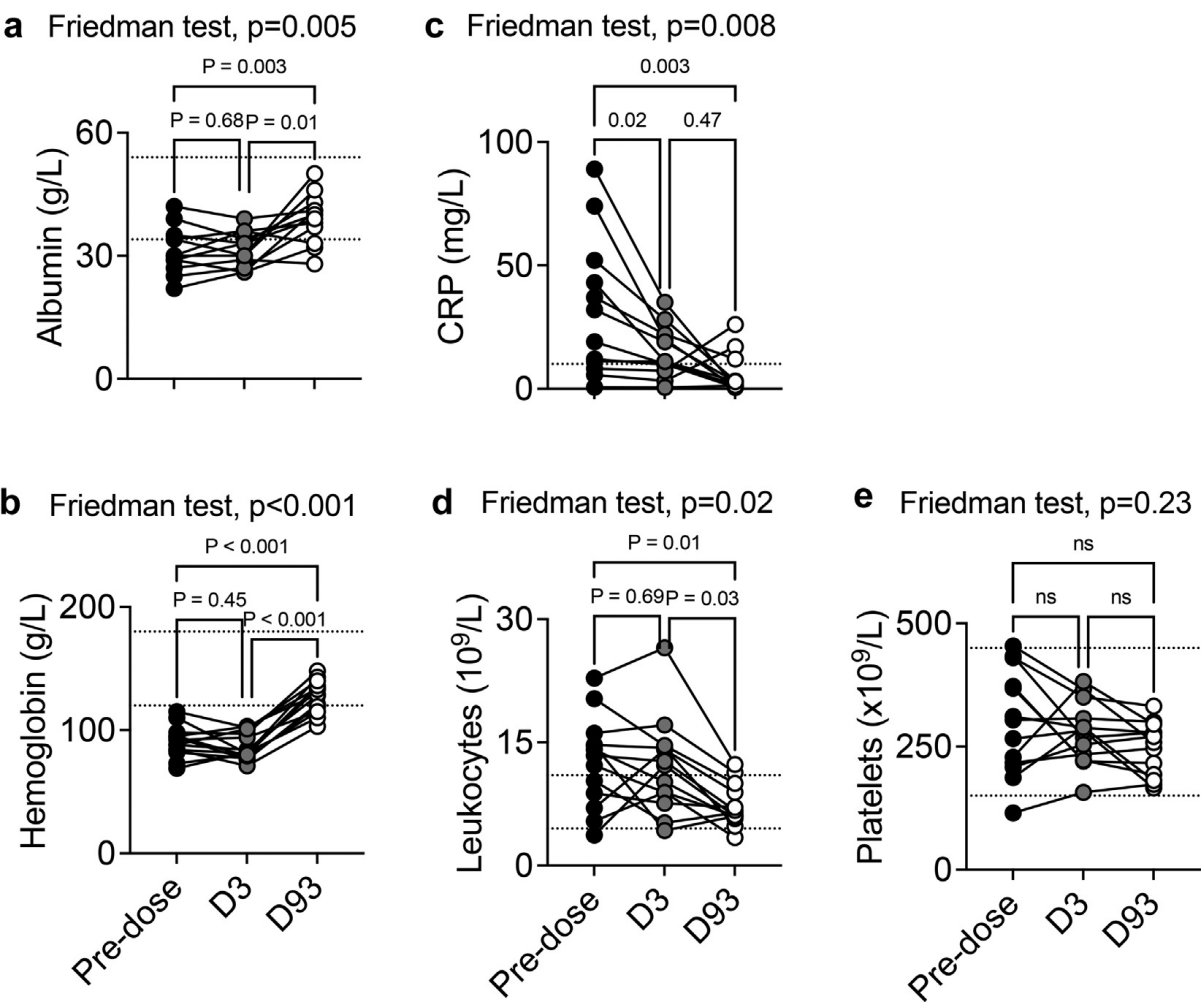
Improvement of the biological parameters in patients from the GOOD-IDES-01 trial after treatment (standard care + Imlifidase). Analysis was performed on blood samples obtained before as well as 3 and 93 days after standard care and imlifidase treatment. (a) Albumin, (b) hemoglobin, (c) CRP, (d) leukocytes, and (e) platelet counts were studied. Dotted lines indicate normal ranges: albumin: 34–54 g/l, hemoglobin: 120–180 g/l, CRP: >10 mg/l, Leukocytes: 4.5–11 × 10^9^/l, platelets: 150–450 × 10^9^/l. *n* = 12–13 patients per test. Friedman test, followed by uncorrected Dunn’s test. CRP, c-reactive protein; D3, day 3; D93, day 93.

Serum albumin and hemoglobin significantly increased toward normal range values with the treatment, suggesting that disease activity was attenuated (Figure 6a and b). Plasma c-reactive protein and circulating leukocytes were significantly reduced after the treatment, demonstrating that systemic inflammation and leukocyte mobilization were reduced (Figure 6c and d). Finally, even if we only observed a trend in the reduction of the platelet count, by the treatment, on day 93 (Figure 6e), circulating platelet counts were reduced to some extends, over the course of the study, in patients with high platelet counts (Figure 2a).

### IgG Degradation and Immunosuppressive Treatment Does not Control Chronic Platelet Activation in Patients From the GOOD-IDES-01 Trial

**Figure 7:**
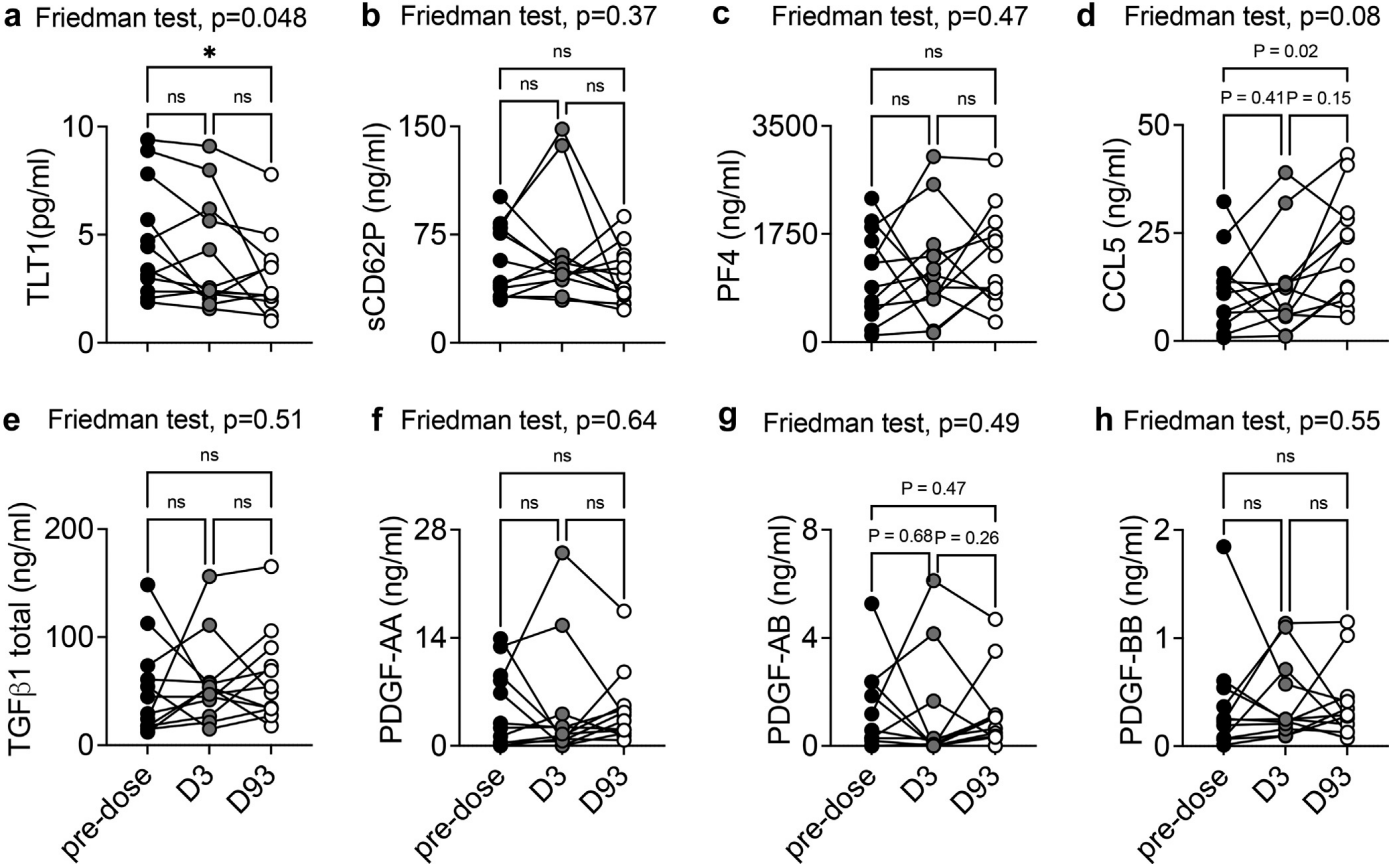
Evolution of the circulating level of platelet activation markers in patients from the GOOD-IDES-01 trial after treatment (standard care + imlifidase). Plasma samples were obtained before, and 3 and 93 days after treatment with standard care and imlifidase. (a) TLT1, (b) soluble CD62P, (c) PF4, (d) CCL5, (e) PDGF-AB, (f) PDGF-BB, (g) PDGF-AA were titrated in the plasma using ELISA. *n* =12 patients per test. Friedman test, followed by uncorrected Dunn’s test. CCL5, C-C motif ligand 5; D3, day 3; D93, day 93; ELISA, enzyme-linked immunosorbent assay; PDGF, platelet-derived growth factor; PF4, platelet factor 4; TGFβ1, transforming growth factor beta 1; TLT1, TREM-like transcript 1.

To understand if the removal of anti-GBM antibodies could reduce platelet activation, we investigated the level of platelet activation markers after the treatment with imlifidase. We found that the treatment significantly reduced plasma Tlt1 (Figure 7a). However, plasma sCD62P and other platelet-derived products were not affected and remained elevated in the plasma until day 93 (Figure 7a–h). This result suggests that Tlt1 could be a more sensitive marker to evaluate chronic platelet activation, as compared with the other platelet-derived products. Endothelial cells can also release sCD62P in the plasma, because it is stored in their Weibel-Palade bodies.^34^ Similarly, PF4, CCL5 TGFβ, and PDGFs are not exclusively expressed by platelets, and may be secreted by other cell types. Furthermore, despite the significant reduction of plasma Tlt1, its concentration remained higher at day 93 than in HBDs (HBDs: 0.77 ng/ml vs. anti-GBM: 3.05 ng/ml, ∗*P* < 0.05), suggesting that chronic platelet activation was not abrogated by the treatment.

### HGFs are Treatment-Sensitive Biomarkers in Patients From the GOOD-IDES-01 Trial

**Figure 8:**
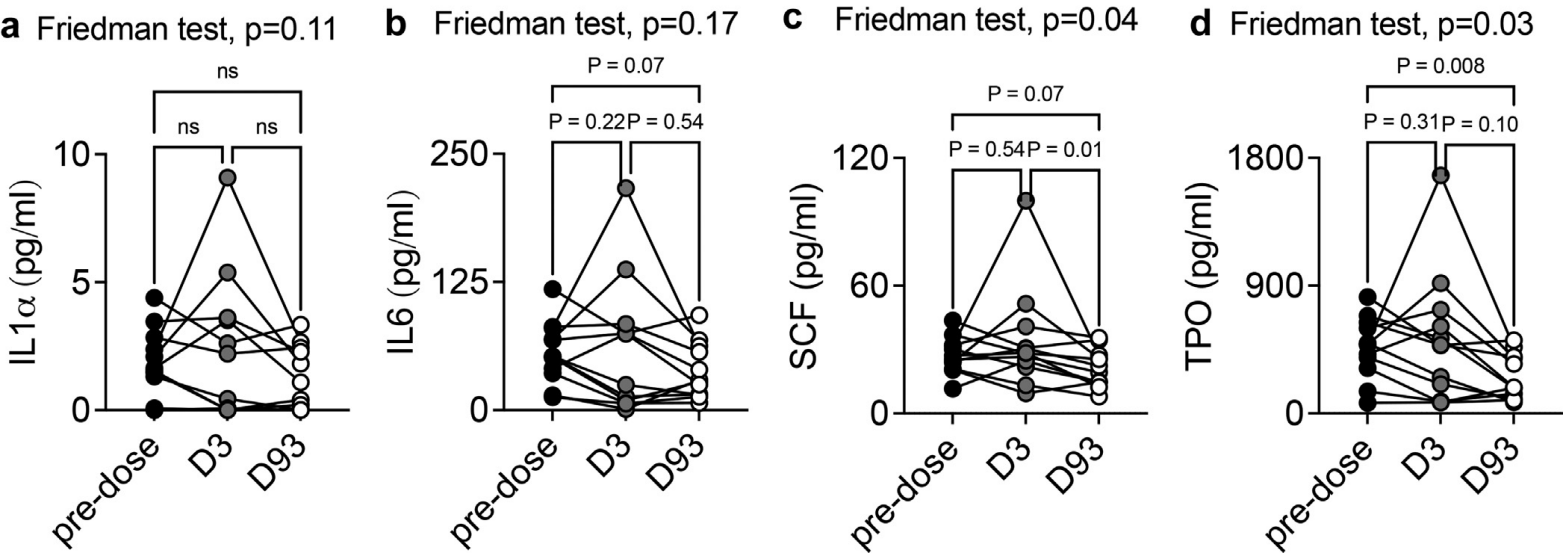
Evolution of the circulating level of pro-inflammatory cytokines and HGFs in patients from the GOOD-IDES-01 trial after the treatment (standard care + imlifidase). Plasma samples were obtained before, and 3 and 93 days after treatment with standard care and Imlifidase. (a) IL1α, (b) IL6, (c) SCF, and (d) TPO were titrated in the plasma using a multiplex analysis. *n* = 12 patients per test. Friedman test, followed by uncorrected Dunn’s test. D3, day 3; D93, day 93; IL, interleukin; SCF, stem cell factor; TPO, thrombopoietin.

Because we observed that proinflammatory cytokines and HGFs stimulate MKpoiesis and thrombopoiesis in experimental anti-GBM disease, we analyzed the evolution of their plasma concentrations following the treatment. Surprisingly, standard care and imlifidase did not significantly reduce the level of circulating IL1α or IL6 (Figure 8a and b). However, we found a significant reduction of both SCF and TPO following the treatment, on day 93 (Figure 8c and d), suggesting that immunosuppression and IgG cleavage can affect the production of HGFs during anti-GBM disease. This result is in line with the reduction of circulating platelet counts, as well as with the significant reduction of platelet activation (plasma Tlt1) following the treatment.

### Potential Prognostic Value of Platelet Activation Markers, Platelet Counts, Platelet-Derived Molecules, Proinflammation Cytokines, and HGFs

To know if the biomarkers that we identified are prognostic biomarkers, and represent tools to predict response to treatment, we analyzed their association with parameters of disease improvement. First, we identified patients who (i) did not required dialysis (*n* = 5); (ii) became independent of dialysis following the treatment with standard care and imlifidase (*n* = 6); (iii) died, or remained dependent on dialysis at the end of the study (*n* = 4). Among these 3 groups, high platelet count and low plasma level of CCL5 at the time of hospitalization (day 0 before imlifidase treatment) tended to be associated with patient becoming dialysis independent, despite the low number of patients in each group (Supplementary Table S2, Supplementary Figure S1A and B). Next, we analyzed the association of our biomarkers with the resolution of glomerular injury, renal insufficiency, and anemia, using surrogates, that is, serum albumin (patients with albumin below normal range [≤ 34 g/l] were selected; Δalbumin), serum creatinine and urea (Δcreatinine, Δurea) and blood hemoglobin concentration (Δhemoglobin), respectively. The improvement was calculated as the difference between day 0 (before imlifidase treatment) and day 93 (after imlifidase treatment). Overall, only the plasma concentration of PDGF-AB and PF4 were positively and significantly associated with Δalbumin (Supplementary Table S3, Supplementary Figure S1C and D). Therefore, we speculated that higher levels of these platelet-related molecules at the beginning of the study is associated with a better resolution of glomerular injury following standard care and imlifidase treatment. These results need to be further confirmed in future studies.

## Discussion

We recently showed that thrombo-inflammation occurs in glomeruli from patients with extracapillary glomerulonephritis.^25^ This new study confirms that thrombo-inflammation is chronically activated in patients with anti-GBM disease. As anti-GBM autoantibodies specifically target glomeruli, we can infer that chronic glomerular thrombo-inflammation is a hallmark of the disease pathophysiology. Anti-GBM autoantibodies might trigger thrombo-inflammation in the initial phase of the disease. However, the chronic thrombo-inflammatory process seems perpetuated by other mechanisms, independent of the direct recognition of glomerular anti-GBM antibodies by platelets, given that platelet activation markers remained elevated in the plasma of patients treated with standard care and Imlifidase. Importantly, our results indicate that platelet activation should be monitored closely because of the large amount of proinflammatory, promitotic, and profibrotic factors that they can release, and given the therapeutic arsenal that we have at our disposal to control these cells.

Antiplatelet therapies could be used to prevent chronic platelet activation, and to reduce microvascular inflammation and pathological glomerular tissue remodeling. Unspecific platelet inhibition is avoided in anti-GBM disease because it could promote excessive bleeding in patients with pulmonary hemorrhage. However, targeting the specific pathological platelet activating–related pathways could be beneficial for long-term renal function and patient care. Further studies will be required to identify these pathways. Similarly, targeting platelet-derived molecules could be beneficial to improve disease outcome. We highlighted that plasma levels of PF4, CCL5, TGFβ, and PDGFs are strongly associated with platelet activation. Because they could be released in glomeruli during the active phase of the disease, developing antagonizing strategies against these biologically active molecules could prove efficient in patients with anti-GBM disease.

As previously observed in experimental anti-GBM disease and in patients with antineutrophil cytoplasmic autoantibody vasculitis–associated glomerulonephritis,^25^ the increased bioavailability of plasma HGFs, TPO and SCF; and proinflammatory cytokines, IL1α and IL6, is also a hallmark of anti-GBM disease in patients. This increase could boost MKpoiesis and platelet production. Because TPO neutralization in experimental anti-GBM disease normalized platelet production, significantly reduced chronic thrombo-inflammation, and improved kidney disease outcome, this new set of data strongly support the idea that TPO neutralization could also improve disease outcome in patients with anti-GBM disease and elevated platelet count, on top of standard care treatment.

Overall, platelets, their derived products, and HGFs promoting MKpoiesis are potential targets in anti-GBM disease that deserve further attention.

### Platelet Activation and New Therapeutic Opportunities in Anti-GBM Disease

Traditional therapies targeting platelet, thrombosis, and coagulation (clopidogrel, aspirin, tirofiban, varoxapar, and dabigatran) inhibit amplification signals (ADP and thromboxane A2), the binding of activated GpIIbIIIa to fibrinogen, and the thrombin-PAR1 pathway, respectively. Despite their potency, their use is precluded to avoid hemorrhage in anti-GBM. However, platelets can be activated by various ligand-receptor interactions, including collagen-GPVI, podoplanin-Clec2, and vWF-GpIb-V-IX.

The inhibition of GPVI, in experimental anti-GBM disease, prevented platelet accumulation in glomeruli, suggesting that GPVI enhances glomerular thrombo-inflammation.^24^ Recently, a GPVI-targeting antibody (glenzocimab) was developed to prevent platelet activation. This strategy efficiently prevents thrombo-inflammation in the brain during acute ischemic stroke, without causing any bleeding disorder.^35^ This innovative treatment could be considered in patients developing anti-GBM disease, because the GPVI pathway seems involved in the disease physiopathology.

Platelet-induced glomerular recruitment of neutrophils is instrumental in initiating thrombo-inflammation during experimental anti-GBM disease, and rely on platelet CD62P.^24^ Recently, a monoclonal anti-CD62P–neutralizing antibody (inclacumab) was developed. It is safe and efficient to prevent platelet-leukocyte aggregates, without affecting platelet count, or bleeding time.^36^ Preventing neutrophil-platelet and platelet-endothelium interactions by blocking CD62P could alleviate glomerular thrombo-inflammation in patients with anti-GBM disease. These new therapeutical opportunities could improve the renal outcome of patients with anti-GBM disease, on top of the newly developed imlifidase and immunosuppressive treatment.

### A Better Understanding of MKpoiesis During Inflammatory Conditions to Improve Platelet Quality

Improving our understanding of MKpoiesis and platelet production in patients with anti-GBM disease is crucial. MKpoiesis is sensitive to inflammatory mediators, which are suspected to skew MKpoiesis toward the production of platelets with an altered phenotype, as previously observed in models of acute inflammation,^37^ sepsis,^38^ and COVID-19.^39^ We showed that IL6 regulates TPO production in experimental anti-GBM disease,^25^ and we observed that platelets produced during experimental anti-GBM disease exhibit a proinflammatory and profibrotic phenotype (unpublished data). In this context, the inflammation-induced production of kidney-derived HGFs precedes MKpoiesis and platelet production. However, this continuum between the inflammatory phase of the disease, the production of kidney-derived HGFs, the activation of Mkpoiesis, and platelet overproduction is almost impossible to catch in small cohorts of patients, with only a few samples. Better understanding of how inflammation reshapes MKpoiesis could refine therapeutic strategies targeting the production of platelets. Restoring normal, or even enhancing, platelet characteristics under chronic inflammatory conditions, could ultimately improve the disease outcome. Because puncturing bone marrow in patients with anti-GBM disease to study the molecular cues at play during MKpoiesis is not desirable, platelet parameters (count, mean platelet volume, and quantity of RNA) and new “omic” technologies (transcriptomic and proteomic) could be used as proxies to assess the activity and the quality of the platelet production in patients, in future studies.

This study has limitations. Owing to the unmatched timing of blood and biopsy sampling, noncentralized measurement of blood cell counts and the small cohort size, this *post hoc* study of patients from the GOOD-IDES-01 trial could not conclusively establish correlations between platelet-derived products, platelet counts, and HGFs; and histological findings from biopsies. The fact that immunosuppressive treatments and plasma exchange had been started in most patients before the first blood samples were drawn most probably attenuated the results presented here and might have blurred correlations. Similarly, we cannot exclude that our observations were affected by other factors related to hospitalization, which include, but are not limited to, malnutrition, repeated blood draws, intraalveolar hemorrhage, hemorrhagic complications from kidney biopsy, erythropoietin administration, or blood transfusion.

The specificity of the increased platelet count, thrombo-inflammatory process, bioavailability of platelet-derived products, and circulating HGFs to the anti-GBM disease was not assessed in our study, because the control group used were HBDs. Investigating if these processes are also enhanced in noninflammatory glomerular diseases, such as diabetic nephropathy, or minimal change disease without autoantibodies, would give important clues about the generalizability of our findings. This aspect will require further studies.

Although some biomarkers were sensitive to treatment in this small cohort, studying a larger cohort, such as GOOD-IDES2 (clinicaltrial.gov; NCT05679401; expected *n* = 50), with 2 arms, standard care with and without imlifidase, might emphasize the importance of studying platelet activation markers, platelet-derived products, and HGFs in anti-GBM disease, and definitively establish their value as treatment-sensitive biomarkers.

Despite the small size of this cohort, we can affirm that platelet count does not reflect platelet activation, and the bioavailability of MKpoiesis-boosting HGFs significantly increases during anti-GBM disease. A more detailed analysis of kidney biopsies could have improved the sensitivity of this study. In experimental anti-GBM disease, glomerular cell proliferation (PCNA^+^ cells), glomerular inflammation (CD45^+^ cells) and TGFβ-induced fibrosis (nuclear accumulation of pSMAD2) are promoted by chronic platelet activation.^25^ However, biopsies in the GOOD-IDES-01 were collected, prepared for analysis and initially analyzed at each local center before being sent to the study pathologists who performed the histological analysis. At this point, additional stainings were not possible. Further studies, including clinical evaluations of new antiplatelet therapies, will be needed to decipher if neutralizing platelet-derived effector molecules can alleviate anti-GBM disease progression toward ESRD. Similarly, the therapeutic effect of targeting TPO and other HGFs in anti-GBM disease should be evaluated.

The GOOD-IDES01 trial has some merits, most importantly that it is the first study to prospectively collect clinical data, blood samples, and biopsies in anti-GBM patients. Therefore, this cohort represents a unique possibility to study the pathophysiology of anti-GBM disease, despite the small sample size.

## Conclusion

Overall, our study demonstrates that the pathophysiology of anti-GBM disease is characterized by chronic thrombo-inflammation and an increased bioavailability of HGFs. Based on our data, we conclude that chronic thrombo-inflammation is highly refractory to immunosuppressive treatment combined to imlifidase, despite the improvement of patients following the treatment.^9^ However, we identified that TPO, SCF, and Tlt1 are treatment-sensitive biomarkers in patients with anti-GBM disease. These findings highlight the potential for these biomarkers to guide more effective therapeutic strategies and improve patient outcomes in the future.

## Disclosure

MS reports being a consultant for Astra Zeneca, Hansa Biopharma and Viafor pharma. IMB reports being a consultant for Hansa Biopharma. All the other authors declared no competing interests.

## Supplementary Material

Supplementary (File (PDF) **Supplementary Methods. Figure S1.** Parameters associated with a good response to treatment. **Table S1**. Correlation matrix. **Table S2.** Descriptive table of covariates, stratified by Δ dialysis. **Table S3.** Spearman rank correlation test between covariates and continuous outcomes. STROBE Checklist.
