# Supplementary material for: Persistent Chronic Thrombo-Inflammation in Anti–Glomerular Basement Membrane Disease Despite Immune Complex Removal
Source: Kidney Int Rep. 2025 Jun 19;10(9):3138–49. doi: 10.1016/j.ekir.2025.06.014 (PMC12446871; doi:10.1016/j.ekir.2025.06.014)
Supplement: Supplementary (File (PDF) — Supplementary Methods. Figure S1. Parameters associated with a good response to treatment. Table S1. Correlation matrix. Table S2. Descriptive table of covariates, stratified by Δ dialysis. Table S3. Spearman rank correlation test between covariates and continuous outcomes. STROBE Checklist. [file mmc1.pdf]

Supplemental methods.

Histological and biological parameters

For the purpose of this study, histological data used included the percentage of cellular crescents, of fibrous crescents, of sclerotic crescents, and of interstitial fibrosis and tubular atrophy (IFTA), all assessed by two separate renal pathologists as part of the original trial[9].

Anti-GBM levels in serum was measured at the Department of Clinical Immunology and Transfusion Medicine, Region Östergötland, Linköping, Sweden. Hemoglobin, leukocyte and platelet counts were measured at the local hospitals during the trial. CRP, albumin, urea and creatinine for eGFR calculations were measured at the Department of Clinical Chemistry, University and Regional Laboratories, Region Skane, Lund, Sweden, and eGFR were calculated based on serum creatinine using the Modification of Diet in Renal Disease formula. For all calculations in this study, eGFR was set to 0 mL/min/1.73 m2 for dialysis-dependent patients and the patient who died anuric during the study. However, for one patient who only required two dialysis sessions per week at 6 months and could stop dialysis at 8 months, eGFR was arbitrarily set to 5 mL/min/1.73 m2.

Titration of human pro-inflammatory cytokines, hematopoietic growth factors, platelet activation markers, and platelet-derived products

Plasma samples from patients with anti-GBM disease and HBDs were sawed, centrifuged at 10,000g for 10 min, and 10 µl of undiluted supernatants were used to perform the titration of IL6, IL1α, TPO and SCF, using a bead-based multiplex analysis kit (Thermofisher, Procarta Plex^{TM}) as previously described[25]. Samples were analyzed on a Bioplex 200 analyzer (Luminex technology).

PBS-diluted plasma samples (1/200) were used to titrate platelet activation markers, soluble CD62P and Tlt1, as well as platelet-derived products, PF4, CCL5, and PDGFs (-AA, -AB, -BB), using ELISA kits, according to the manufacturer's instructions (R&D system, DuoSet, [CD62P, cat n° DY137; Tlt1, cat n° DY2394; PF4, cat n° DY795; CCL5, cat n° DY278-05; PDGF-AA, cat n° DY221; PDGF-AB, cat n° DY222; PDGF-BB, cat n° DY220).

For total plasma TGFβ1 titration, samples were first acidified with HCL, for 10 min. Acidification was neutralized using NaOH. Activated plasma samples were used to titrate TGFβ1 using a bead-based kit, according to the manufacturer's instructions

(Thermo Fisher, ProcartaPlex™ Simplex Kit, cat n°EPX01A-20608-901). Samples were analysed on a Bioplex 200 analyser (Luminex).

Statistical analysis

Graphics were generated using Prims10 (GraphPad). Mann-Whitney test was performed to compare 2 groups (n≤15 patients/group). The Friedman test followed by uncorrected Dunn's test was used to compare the evolution of markers following the treatment in patients with anti-GBM disease (n=13). The matrix of correlation, the spearman coefficient table and the probability table were performed using JMP17.

A p value ≤0.05 was considered statistically significant, and is represented in the graphs, as follow: *p<0.05, **p<0.01, ***p<0.001.

Prognostic value of biomarkers.

Continuous data were presented as mean ± standard deviation (SD), and categorical data were reported as counts and percentages (n, %).

Continuous outcomes included albumin, hemoglobin, creatinine, and urea, calculated as the difference between measurements at day 0 and day 93. For Δalbumin analysis, only patients with serum albumin lower than normal range (≤34g/L) were included.

Continuous covariates included CD62P, PF4, TLT-1, Total TGF-β1, PDGF-AA, PDGF-BB, PDGF-AB, CCL5, TPO, SCF, IL6, IL1α and platelet count. Each covariate was evaluated at day 0.

Associations between covariates and dialysis outcome on day 93 (never on dialysis, became independent of dialysis, remained dependent on dialysis) were evaluated using Kruskal-Wallis test followed by uncorrected Dunn's test. Associations between covariates and other continuous outcomes (Δ albumin, Δ hemoglobin, Δ creatinine, Δ urea) were assessed using the Spearman rank correlation test. Significant correlations are represented in **Supplemental figure 1B-E**. Statistical significance was defined as a p-value <0.05.

Statistical analyses were performed using the open-source Python 3.11.4 software package.

Supplemental table 1. Correlation matrix

Probability	Cell crescents (%)	Fibrous Crescents (%)	Global sclerosis (%)	Interstitial fibrosis and tubular atrophy (% IFTA)	Haemoglobin (g/L)	Alb (g/L)	Urea (mmol/L)	eGFR (ml/min/1.73m2)	CRP (mg/L)	Anti-GBM (U/mL)	Leucocytes (x10^9)	Thrombocytes (x10^9/L)	IL1α (pg/ml)	IL6 (pg/ml)	SCF (pg/ml)	TPO (pg/ml)	PDGFAA (ng/ml)	PDGFBB (ng/ml)	PDGFAB (ng/ml)	CCL5 (ng/ml)	CD62P (ng/ml)	PF4 (ng/ml)	TLT-1 (ng/ml)	Total TGFβ (ng/ml)
Cell crescents (%)		0,1035	0,274	0,0076*	0,0150*	0,8893	0,7078	0,846	0,4498	0,31	0,936	0,6747	0,3288	0,5171	0,5171	0,2834	0,9641	0,6914	0,8979	0,8754	0,8052	0,7306	0,4268	0,2876
Fibrous Crescents (%)			0,549	0,1813	0,0548	0,2556	0,5013	0,7315	0,8947	0,7315	0,8758	0,9752	0,9596	0,7731	0,5781	0,9162	0,1889	0,188	0,2365	0,2352	0,0991	0,3799	0,3843	0,4163
Global sclerosis (%)				0,0188*	0,8922	0,2299	0,0412*	0,8101	0,7811	0,4202	0,5522	0,4809	0,9521	0,988	0,1615	0,545	0,3531	0,135	0,2498	0,4112	0,2088	0,2964	0,2211	0,3849
Interstitial fibrosis and tubular atrophy (% IFTA)					0,9457	0,7202	0,4125	0,5279	0,5329	0,9099	0,0701	0,5028	0,9579	0,8563	0,5279	0,9609	0,9457	0,5638	0,8427	0,7166	0,6168	0,6276	0,6605	0,231
Haemoglobin (g/L)						0,0386*	0,7363	0,5793	0,0694	0,3231	0,0223*	0,7318	0,6583	0,4264	0,6378	0,4672	0,868	0,7462	0,7962	0,9596	0,8195	0,9042	0,6424	0,9243
Alb (g/L)							0,7475	0,3552	0,2024	0,6002	0,0398*	0,5259	0,9518	0,7164	0,3693	0,794	0,4243	0,9341	0,4277	0,4679	0,6597	0,4286	0,5387	0,9291
Urea (mmol/L)								0,0207*	0,5621	0,3684	0,4001	0,4664	0,2282	0,2769	0,2314	0,0503	0,9441	0,2983	0,5685	0,8495	0,9697	0,6568	0,8694	0,3544
eGFR (ml/min/1.73m2)									0,6848	0,7613	0,8286	0,5413	0,0515	0,1961	0,7905	0,2314	0,9543	0,3618	0,8267	0,9798	0,9396	0,9195	0,8595	0,6479
CRP (mg/L)										0,065	0,2628	0,0192*	0,5363	0,435	0,5581	0,4193	0,6446	0,9899	0,8873	0,8198	0,3791	0,9899	0,0082*	0,7036
Anti-GBM (U/mL)											0,9228	0,5327	0,2739	0,451	0,3688	0,0188*	0,7739	0,8994	0,56	0,6205	0,8595	0,6945	0,1598	0,4201
Leucocytes (x10^9)												0,5733	0,1687	0,0694	0,5426	0,9583	0,4266	0,9346	0,4445	0,5325	0,8991	0,4833	0,852	0,9941
Thrombocytes (x10^9/L)													0,6202	0,8298	0,742	0,6476	0,6682	0,771	0,9692	0,9899	0,216	0,9899	0,0172*	0,8003
IL1α (pg/ml)														0,0004*	0,0068*	0,0139*	0,6331	0,7708	0,6936	0,6339	0,6522	0,7036	0,3614	0,6801
IL6 (pg/ml)															0,0272*	0,0049*	0,0324*	0,0867	0,0609	0,0261*	0,0736	0,0428*	0,0924	0,0642
SCF (pg/ml)																0,0242*	0,9746	0,8795	0,9692	0,9698	0,8595	0,8101	0,8199	0,8298
TPO (pg/ml)																	0,6079	0,5756	0,9692	0,5239	0,6248	0,6384	0,3649	0,6476
PDGFAA (ng/ml)																		<,0001*	<,0001*	<,0001*	<,0001*	<,0001*	0,0176*	0,0003*
PDGFBB (ng/ml)																			<,0001*	<,0001*	0,0002*	<,0001*	0,0498*	0,0001*
PDGFAB (ng/ml)																				<,0001*	<,0001*	<,0001*	0,0400*	0,0002*
CCL5 (ng/ml)																					<,0001*	<,0001*	0,0687	<,0001*
CD62P (ng/ml)																						<,0001*	0,0002*	0,0002*
PF4 (ng/ml)																							0,0412*	<,0001*
TLT-1 (ng/ml)																								0,0517
Total TGFβ (ng/ml)																								

Spearman coefficient	Cell crescents (%)	Fibrous Crescents (%)	Global sclerosis (%)	Interstitial fibrosis and tubular atrophy (% IFTA)	Haemoglobin (g/L)	Alb (g/L)	Urea (mmol/L)	eGFR (ml/min/1.73m2)	CRP (mg/L)	Anti-GBM (U/mL)	Leucocytes (x10^9)	Thrombocytes (x10^9/L)	IL1α (pg/ml)	IL6 (pg/ml)	SCF (pg/ml)	TPO (pg/ml)	PDGFAA (ng/ml)	PDGFBB (ng/ml)	PDGFAB (ng/ml)	CCL5 (ng/ml)	CD62P (ng/ml)	PF4 (ng/ml)	TLT-1 (ng/ml)	Total TGFβ (ng/ml)
Cell crescents (%)		-0,4534	-0,3142	-0,6786	-0,6336	-0,0410	0,1101	-0,0572	0,22	0,2926	-0,0248	0,1232	0,2819	0,1892	0,1892	0,3084	-0,0133	0,1166	-0,0378	0,0462	0,0726	0,1012	0,231	0,3058
Fibrous Crescents (%)			0,1752	0,3791	0,5234	0,3258	-0,1963	-0,1009	-0,0390	0,1009	0,0482	0,0092	0,0149	0,0848	0,1628	0,031	0,3731	0,3738	0,3385	0,3394	0,4586	0,2545	0,2522	0,2362
Global sclerosis (%)				0,6168	-0,0399	0,343	-0,5509	-0,0707	-0,0818	-0,2343	-0,1818	-0,2055	0,0177	0,0044	0,3956	0,177	-0,2686	-0,4199	-0,3296	-0,2387	-0,3580	-0,3006	-0,3492	-0,2519
Interstitial fibrosis and tubular atrophy (% IFTA)					0,0201	-0,1053	-0,2381	-0,1845	0,1822	0,0333	-0,5174	-0,1956	-0,0156	0,0533	0,1845	0,0145	-0,0201	-0,1689	-0,0584	-0,1067	-0,1467	-0,1422	-0,1289	-0,3422
Haemoglobin (g/L)						0,5378	0,095	0,1558	-0,4812	-0,2739	0,6035	-0,0967	-0,1246	-0,2220	-0,1325	-0,2034	0,047	0,0913	0,0729	-0,0143	0,0645	-0,0340	-0,1307	-0,0269
Alb (g/L)							0,0908	-0,2570	-0,3489	-0,1474	0,5541	-0,1779	0,0171	-0,1024	0,2498	0,0737	-0,2230	-0,0234	-0,2214	-0,2031	-0,1240	-0,2210	-0,1725	0,0252
Urea (mmol/L)								-0,5898	-0,1628	-0,2502	0,2442	-0,2038	-0,3309	-0,3003	-0,3289	-0,5134	0,0198	0,2878	0,1602	0,0536	0,0107	0,1251	-0,0465	0,2574
eGFR (ml/min/1.73m2)									-0,1144	0,0857	-0,0637	0,1714	0,5112	0,3536	0,075	0,3289	0,0162	-0,2536	-0,0618	-0,0071	0,0214	-0,0286	0,05	-0,1286
CRP (mg/L)										0,4879	-0,3212	0,5952	0,1735	0,2181	0,1644	0,2254	0,1299	0,0036	0,04	-0,0643	0,2449	-0,0036	0,6542	-0,1072
Anti-GBM (U/mL)											-0,0286	0,175	0,3021	0,2107	0,25	0,597	-0,0811	0,0357	-0,1637	-0,1393	0,05	-0,1107	0,3821	-0,2250
Leucocytes (x10^9)												0,1648	-0,3894	-0,4989	-0,1780	-0,0154	-0,2311	-0,0242	-0,2225	-0,1824	-0,0374	-0,2044	-0,0549	-0,0022
Thrombocytes (x10^9/L)													-0,1394	0,0607	-0,0929	0,1287	0,1207	-0,0821	-0,0109	-0,0036	0,3393	0,0036	0,6036	0,0714
IL1α (pg/ml)														0,7989	0,6649	0,619	0,1344	0,0822	0,111	0,134	0,1269	0,1072	0,2538	0,1162
IL6 (pg/ml)															0,5679	0,6845	0,5532	0,4571	0,4946	0,5714	0,475	0,5286	0,45	0,4893
SCF (pg/ml)																0,5773	-0,0090	0,0429	-0,0109	-0,0107	-0,0500	-0,0679	0,0643	0,0607
TPO (pg/ml)																	0,1443	0,1573	0,0109	0,1787	0,1376	0,1323	0,252	0,1287
PDGFAA (ng/ml)																		0,8919	0,9725	0,9478	0,9081	0,9658	0,6018	0,8054
PDGFBB (ng/ml)																			0,9202	0,8964	0,8179	0,9143	0,5143	0,8286
PDGFAB (ng/ml)																				0,942	0,8656	0,9602	0,5346	0,8183
CCL5 (ng/ml)																					0,8571	0,9786	0,4821	0,8893
CD62P (ng/ml)																						0,875	0,8214	0,8179
PF4 (ng/ml)																							0,5321	0,8857
TLT-1 (ng/ml)																								0,5107
Total TGFβ (ng/ml)																								

Supplemental table 2: descriptive table of covariates, stratified by Δ dialysis

	Overall *Mean (SD)*	Never on dialysis *Mean (SD)*	Quite dialysis *Mean (SD)*	Remained on dialysis *Mean (SD)*	p-value
**N=**	**15**	**5**	**6**	**4**	
**sCD62P (ng/ml)**	*54.1 (23.4)*	*51.55 (19.85)*	*50.83 (22.64)*	*62.23 (32.58)*	0.8913
**PF4 (ng/ml)**	*1035.0 (756.3)*	*1246 (621.7)*	*708.8 (753.9)*	*1260 (922.9)*	0.3950
**TLT-1 (pg/ml)**	*4512.6 (2461.6)*	*3732 (2942)*	*4844 (2578)*	*4991 (2029)*	0.4274
**Total TGFβ (ng/ml)**	*50.2 (40.8)*	*53.86 (29.73)*	*43.40 (52.85)*	*55.93 (41.96)*	0.5738
**PDGF-AA (ng/ml)**	*4.4 (5.0)*	*5.089 (5.238)*	*2.981 (4.495)*	*5.765 (6.118)*	0.4744
**PDGF-BB (pg/ml)**	*318.8 (460.5)*	*226.9 (104.5)*	*147.1 (210.6)*	*691.3 (802)*	0.1045
**PDGF-AB (ng/ml)**	*1075.4 (1464.4)*	*1032 (959.7)*	*593 (992.5)*	*1853 (2421)*	0.3080
**CCL5 (ng/ml)**	*11.4 (9.8)*	*15.5 (9.383)*	*5.958 (6.371)*	*14.6 (12.88)*	0.2021
**TPO (pg/ml)**	*455.4 (191.0)*	*497.9 (190)*	*424 (150.4)*	*449.3 (281.5)*	0.9626
**SCF (pg/ml)**	*27.0 (7.5)*	*29.48 (8.928)*	*26.59 (4.459)*	*24.66 (10.36)*	0.9005
**IL6 (pg/ml)**	*54.3 (27.0)*	*69.57 (30.69)*	*46.75 (24.27)*	*46.40 (23.81)*	0.4930
**IL1α (pg/ml)**	*1.7 (1.2)*	*2.292 (1.226)*	*1.497 (1.310)*	*1.403 (1.161)*	0.4464
**Platelet count ($x10^9$/L)**	*296.7 (99.2)*	*254 (71.77)*	*350 (71.06)*	*270.3 (144.8)*	0.2021

*P-values marked with * are considered significant. Kruskall-wallis test followed by uncorrected Dunn's test.*

Supplemental table 3: Spearman rank correlation test between covariates and continuous outcomes

Covariate	Δ albumin	Δ creatinine	Δ hemoglobin	Δ urea
**Platelet count ($x10^9$/L)**	0.635	0.618	0.788	0.803
**CCL5 (ng/ml)**	0.072	0.762	0.115	0.384
**sCD62P (ng/ml)**	0.114	0.914	0.378	0.721
**IL1α (pg/ml)**	0.902	0.058	0.849	0.121
**IL6 (pg/ml)**	0.524	0.095	0.382	0.264
**PDGF-AA (ng/ml)**	0.086	0.803	0.107	0.409
**PDGF-AB (ng/ml)**	**0.049***	0.828	0.198	0.236
**PDGF-BB (pg/ml)**	0.081	0.897	0.313	0.170
**PF4 (ng/ml)**	**0.047***	0.746	0.066	0.307
**SCF (pg/ml)**	0.553	0.846	0.828	0.280
**TLT-1 (pg/ml)**	0.213	0.602	0.599	0.795
**TPO (pg/ml)**	0.121	0.158	0.408	0.072
**Total TGFβ (ng/ml)**	0.081	0.779	0.361	0.409

*P-values marked with * are considered significant.*

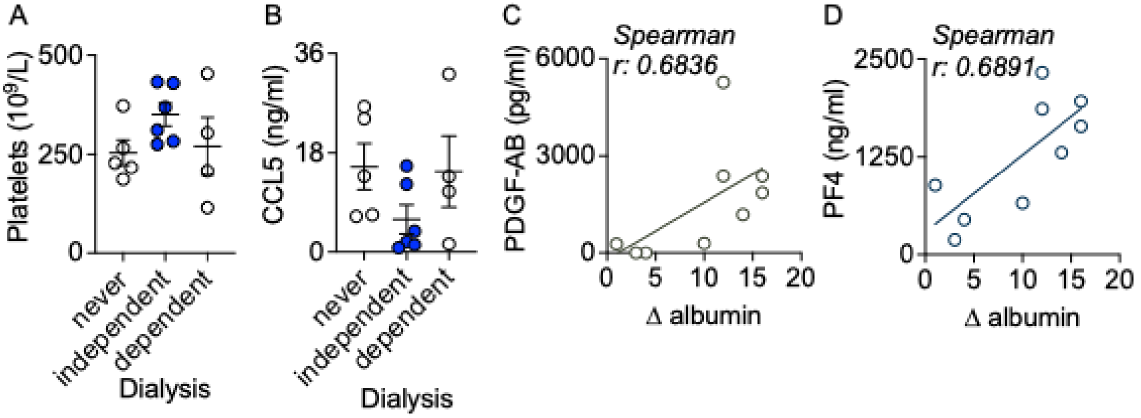

Supplemental Figure 1: Parameters associated with a good response to treatment. Platelet count (**A**) and plasma CCL5 (**B**) of patients enrolled in GOOD-IdeS-01 trial at day 0 (pre-treatment) depending on their group of dialysis on day 93 (never on dialysis, out of dialysis, dialysis dependent). **C, D**. Linear regression analysis of the Δalbumin in patients with low albumin (≤34g/L) before imlifidase treatment with plasma PDGF-AB (**C**) and PF4 (**D;** Δalbumin >0= more serum albumin on day 93 than day 0). Blue circles: patients who became dialysis independent.

STROBE Statement—checklist of items that should be included in reports of observational studies

	Item No.	Recommendation	Page No.	Relevant text from manuscript
**Title and abstract**	1	(*a*) Indicate the study's design with a commonly used term in the title or the abstract	3	**Methods.** In the GOOD-IDES-01 trial, patients received standard care and Imlifidase, containing the IgG-degrading enzyme, IdeS. In plasma samples from patients -collected before and after imlifidase treatment - and healthy blood donors (HBDs), we analyzed plasma HGFs, pro-inflammatory and platelet activation markers, and platelet-derived products.
		(*b*) Provide in the abstract an informative and balanced summary of what was done and what was found	3	**Results.** Anti-GBM disease significantly elevated plasma pro-inflammatory and platelet activation markers, and HGFs (TPO, stem cell factor). Plasma TPO correlated with anti-GBM titers. Standard care and imlifidase treatment only reduced TPO levels and platelet counts. Platelet activation markers (CD62P, Tlt1) and platelet-derived

				products (PDGF, CCL5, PF4, TGFβ), strongly correlated during the active phase of the disease, but remained elevated despite the treatment.
**Introduction**				
Background/rationale	2	Explain the scientific background and rationale for the investigation being reported	5-7	
Objectives	3	State specific objectives, including any prespecified hypotheses	7-8	Given that platelets continuously flow through and accumulate in the glomerular capillary and that anti-GBM autoantibodies could activate platelets via the Fc□RIIA, we hypothesize that this interaction could promote chronic platelet activation and inflammation in glomeruli. In this scenario, removing anti-GBM autoantibodies from glomeruli should quickly stop chronic platelet activation and reduce the level of circulating platelet activation markers, as well as inflammation. Conversely, if removing anti-GBM autoantibodies does not reduce platelet activation markers, it

				would suggest that platelets are activated independently of their presence and Fc□RIIA signaling in patients with established anti-GBM disease. We tested this hypothesis in the context of the GOOD-IDES-01 clinical trial in patients with anti-GBM disease wherein the benefit of removing all IgGs, including glomerular anti-GBM autoantibodies, was evaluated using imlifidase.
**Methods**				
Study design	4	Present key elements of study design early in the paper	8	Briefly, patients with circulating anti-GBM antibodies, and an eGFR<15ml/min/1.73m^2 were included and treated with a single dose of imlifidase in addition to standard therapy according to local guidelines. Plasma samples from patients (n=15) were purified from blood drawn on EDTA and frozen, prospectively for six months. In this study, we analyzed samples at three

				key time points: before treatment with imlifidase (pre-dose), at day 3 and at day 93 post-treatment (**Figure 1**). This enabled us to evaluate the changes in the active phase of the disease, and the rapid and long-term effect of the treatment on HGFs, pro-inflammatory cytokines, and platelet biology, respectively. Control plasma samples were purified from samples of HBDs (n=3) drawn on EDTA, and frozen.
Setting	5	Describe the setting, locations, and relevant dates, including periods of recruitment, exposure, follow-up, and data collection	8	The GOOD-IDES-01 trial (ClinicalTrials.gov: NCT03157037) was previously described[9]
Participants	6	(*a*) *Cohort study*—Give the eligibility criteria, and the sources and methods of selection of participants. Describe methods of follow-up<br>*Case-control study*—Give the eligibility criteria, and the sources and methods of case ascertainment and control selection. Give the rationale for the choice of cases and controls<br>*Cross-sectional study*—Give the eligibility criteria, and the sources and methods of selection of participants	8	The GOOD-IDES-01 trial (ClinicalTrials.gov: NCT03157037) was previously described[9]. Briefly, patients with circulating anti-GBM antibodies, and an eGFR<15ml/min/1.73m^2 were included and treated with a single dose of imlifidase in addition to

standard therapy according to local guidelines. Plasma samples from patients (n=15) were purified from blood drawn on EDTA and frozen, prospectively for six months. In this study, we analyzed samples at three key time points: before treatment with imlifidase (pre-dose), at day 3 and at day 93 post-treatment (**Figure 1**). This enabled us to evaluate the changes in the active phase of the disease, and the rapid and long-term effect of the treatment on HGFs, pro-inflammatory cytokines, and platelet biology, respectively. Control plasma samples were purified from samples of HBDs (n=3) drawn on EDTA, and frozen. Study drugs, treatment regimen, follow-up, laboratory analysis, diagnosis, primary and secondary outcomes, and treatment efficiency validation were all previously described[9].

		(*b*) *Cohort study*—For matched studies, give matching criteria and number of exposed and unexposed<br>*Case-control study*—For matched studies, give matching criteria and the number of controls per case		
Variables	7	Clearly define all outcomes, exposures, predictors, potential confounders, and effect modifiers. Give diagnostic criteria, if applicable	8	The GOOD-IDES-01 trial (ClinicalTrials.gov: NCT03157037) was previously described[9]. Briefly, patients with circulating anti-GBM antibodies, and an eGFR<15ml/min/1.73m^2 were included and treated with a single dose of imlifidase in addition to standard therapy according to local guidelines. Plasma samples from patients (n=15) were purified from blood drawn on EDTA and frozen, prospectively for six months. In this study, we analyzed samples at three key time points: before treatment with imlifidase (pre-dose), at day 3 and at day 93 post-treatment (**Figure 1**). This enabled us to evaluate the changes in the active phase of the disease, and the rapid and long-term effect of the

				treatment on HGFs, pro-inflammatory cytokines, and platelet biology, respectively. Control plasma samples were purified from samples of HBDs (n=3) drawn on EDTA, and frozen. Study drugs, treatment regimen, follow-up, laboratory analysis, diagnosis, primary and secondary outcomes, and treatment efficiency validation were all previously described[9]. To prevent any delay before beginning the treatment, kidney biopsies before inclusion in the trial was not a requirement. Therefore, the timing of the kidney biopsies 9<br><br>does not match the timing of blood sampling and vary between the patients included in the trial.
Data sources/ measurement	8*	For each variable of interest, give sources of data and details of methods of assessment (measurement). Describe comparability of assessment methods if there is more than one group	*8 + supplmental material*	Hemoglobin, leukocyte and platelet counts were measured at the local hospitals during the trial. CRP, albumin, urea and

				creatinine for eGFR calculations were measured at the Department of Clinical Chemistry, University and Regional Laboratories, Region Skane, Lund, Sweden, and eGFR were calculated based on serum creatinine using the Modification of Diet in Renal Disease formula. For all calculations in this study, eGFR was set to 0 mL/min/1.73 m2 for dialysis-dependent patients and the patient who died anuric during the study. However, for one patient who only required two dialysis sessions per week at 6 months and could stop dialysis at 8 months, eGFR was arbitrarily set to 5 mL/min/1.73 m2.
Bias	9	Describe any efforts to address potential sources of bias	Supplemental material	Hemoglobin, leukocyte and platelet counts were measured at the local hospitals during the trial.

				CRP, albumin, urea and creatinine for eGFR calculations were measured at the Department of Clinical Chemistry, University and Regional Laboratories, Region Skane, Lund, Sweden, and eGFR were calculated based on serum creatinine using the Modification of Diet in Renal Disease formula. For all calculations in this study, eGFR was set to 0 mL/min/1.73 m2 for dialysis-dependent patients and the patient who died anuric during the study. However, for one patient who only required two dialysis sessions per week at 6 months and could stop dialysis at 8 months, eGFR was arbitrarily set to 5 mL/min/1.73 m2.
Study size	10	Explain how the study size was arrived at	8	The GOOD-IDES-01 trial (ClinicalTrials.gov: NCT03157037) was

	previously described[9]. Briefly, patients with circulating anti-GBM antibodies, and an eGFR<15ml/min/1.73m^2 were included and treated with a single dose of imlifidase in addition to standard therapy according to local guidelines. Plasma samples from patients (n=15) were purified from blood drawn on EDTA and frozen, prospectively for six months.

Continued on next page

Quantitative variables	11	Explain how quantitative variables were handled in the analyses. If applicable, describe which groupings were chosen and why	Supplemental material	Continuous data were presented as mean ± standard deviation (SD), and categorical data were reported as counts and percentages (n, %).<br>Continuous outcomes included albumin, hemoglobin, creatinine, and urea, calculated as the difference between measurements at day 0 and day 93. Dialysis discharge was analyzed as a binary outcome, defined as 1 if the change in dialysis status from day 0 to day 93 was categorized as "yes-no," indicating cessation of dialysis, and 0 otherwise.<br>Continuous covariates included

				CD62P, PF4, TLT-1, Total TGF-β1, PDGF-AA, PDGF-BB, PDGF-AB, CCL5, TPO, SCF, IL6, IL1α and platelet count. Each covariate was evaluated at day 0.<br>Associations between covariates and Δ dialysis were evaluated using Student's t-test. Associations between covariates and other continuous outcomes (Δ albumin, Δ hemoglobin, Δ creatinine, Δ urea) were assessed using the Spearman rank correlation test.
Statistical methods	12	(*a*) Describe all statistical methods, including those used to control for confounding	Supplemental material	
		(*b*) Describe any methods used to examine subgroups and interactions	NA	
		(*c*) Explain how missing data were addressed	NA	
		(*d*) *Cohort study*—If applicable, explain how loss to follow-up was addressed<br>*Case-control study*—If applicable, explain how matching of cases and controls was addressed<br>*Cross-sectional study*—If applicable, describe analytical methods taking account of sampling strategy	NA	

	(*e*) Describe any sensitivity analyses	Supplemental material	Continuous data were presented as mean ± standard deviation (SD), and categorical data were reported as counts and percentages (n, %).<br>Continuous outcomes included albumin, hemoglobin, creatinine, and urea, calculated as the difference between measurements at day 0 and day 93. Dialysis discharge was analyzed as a binary outcome, defined as 1 if the change in dialysis status from day 0 to day 93 was categorized as "yes-no," indicating cessation of dialysis, and 0 otherwise.<br>Continuous covariates included

				CD62P, PF4, TLT-1, Total TGF-β1, PDGF-AA, PDGF-BB, PDGF-AB, CCL5, TPO, SCF, IL6, IL1α and platelet count. Each covariate was evaluated at day 0. Associations between covariates and Δ dialysis were evaluated using Student's t-test. Associations between covariates and other continuous outcomes (Δ albumin, Δ hemoglobin, Δ creatinine, Δ urea) were assessed using the Spearman rank correlation test.
**Results**				
Participants	13*	(a) Report numbers of individuals at each stage of study—eg numbers potentially eligible, examined for eligibility, confirmed eligible, included in the study, completing follow-up, and analysed	p12	To this end, only patients with paired samples were analyzed (n=12-13).
		(b) Give reasons for non-participation at each stage	NA	
		(c) Consider use of a flow diagram	Figure 1	
Descriptive data	14*	(a) Give characteristics of study participants (eg demographic, clinical, social) and information on exposures and potential confounders	p8	The GOOD-IDES-01 trial (ClinicalTrials.gov: NCT03157037) was

				previously described[9]. Briefly, patients with circulating anti-GBM antibodies, and an eGFR<15ml/min/1.73m[2] were included and treated with a single dose of imlifidase in addition to standard therapy according to local guidelines.
		(b) Indicate number of participants with missing data for each variable of interest	p12	To this end, only patients with paired samples were analyzed (n=12-13).
		(c) *Cohort study*—Summarise follow-up time (eg, average and total amount)	Supplemental material	
Outcome data	15*	*Cohort study*—Report numbers of outcome events or summary measures over time	*P8 + supplemental material*	The GOOD-IDES-01 trial (ClinicalTrials.gov: NCT03157037) was previously described[9].<br><br>. For all calculations in this study, eGFR was set to 0 mL/min/1.73 m2 for dialysis-dependent patients and the patient who died anuric during the study. However, for one patient who only required two dialysis sessions per week at 6 months and could stop dialysis at 8 months, eGFR was arbitrarily set to 5 mL/min/1.73 m2.
		Case-control study—Report numbers in each exposure category, or summary measures of exposure		

		Cross-sectional study—Report numbers of outcome events or summary measures	
Main results	16	(*a*) Give unadjusted estimates and, if applicable, confounder-adjusted estimates and their precision (eg, 95% confidence interval). Make clear which confounders were adjusted for and why they were included	NA
		(*b*) Report category boundaries when continuous variables were categorized	NA
		(*c*) If relevant, consider translating estimates of relative risk into absolute risk for a meaningful time period	NA

Continued on next page

Other analyses	17	Report other analyses done—eg analyses of subgroups and interactions, and sensitivity analyses	NA	
**Discussion**				
Key results	18	Summarise key results with reference to study objectives	14	This new set of data confirms that thrombo-inflammation is chronically activated in the population of patients with anti-GBM disease. As anti-GBM autoantibodies specifically target glomeruli, we can infer that chronic glomerular thrombo-inflammation is a hallmark of the disease pathophysiology. Anti-GBM autoantibodies might trigger thrombo-inflammation in the initial phase of the disease. However, the chronic thrombo-inflammatory process might be perpetuated by other mechanisms, independent of the direct recognition of glomerular anti-GBM antibodies by platelets, given that platelet activation markers remained elevated in the plasma of patients treated with standard care and Imlifidase. Importantly, our results indicate that platelet activation should be monitored closely

				given the large amount of pro-inflammatory, pro-mitotic and pro-fibrotic factors that they can release, and given the therapeutic arsenal that we have at our disposal to control these cells.
Limitations	19	Discuss limitations of the study, taking into account sources of potential bias or imprecision. Discuss both direction and magnitude of any potential bias	16	**Limitations.** Due to the unmatched timing of blood and biopsy sampling, non-centralized measument of blood cell counts and the small cohort size, this post-hoc study of patients from the GOOD-IDES-01 trial could not conclusively establish 17<br><br>correlations between platelet-derived products, platelet counts and HGFs, and histological findings from biopsies. The fact that immunosuppressive treatments and PLX had been started in most patients before the 1st blood samples were drawn most probably attenuated the results presented here and might have blurred correlations.<br>Although some biomarkers were sensitive to treatment in

				this small cohort, studying a larger cohort, such as GOOD-IDES2 (clinicaltrial.gov; NCT05679401; expected n=50), with two arms, standard care with and without Imlifidase, might emphasize the importance of studying platelet activation markers, platelet-derived product and HGFs in anti-GBM disease, and definitively establish their value as treatment-sensitive biomarkers
Interpretation	20	Give a cautious overall interpretation of results considering objectives, limitations, multiplicity of analyses, results from similar studies, and other relevant evidence	P15	This new set of data strongly supports the idea that targeting TPO in patients with anti-GBM disease and elevated platelet count could improve disease outcome. Overall, platelets, their derived products, and HGFs promoting MKpoiesis and platelet production are significant targets in anti-GBM disease and deserve further attention.
Generalisability	21	Discuss the generalisability (external validity) of the study results	P16	Improving our understanding of MKpoiesis and platelet production in patients with chronic inflammatory diseases such as anti-GBM disease is

				crucial. MKpoiesis is sensitive to inflammatory mediators which are suspected to alter MKpoiesis towards the production of platelets with an altered phenotype, as previously observed in models of acute inflammation[37], sepsis[38], and COVID19[39].
**Other information**				
Funding	22	Give the source of funding and the role of the funders for the present study and, if applicable, for the original study on which the present article is based	P20	This work was supported by INSERM (MC), a nonprofit grant from the MSDAvenir fund (SAVE-BRAIN project, GC, AN), Fondation pour la Recherche Médicale (FRM, grant n° ARF201809007140, MC), Laboratory of Excellence INFLAMEX (n° ANR-11-IDEX-0005- 02, MC). LT obtained funding from Thelma Zoégas fund for medical research and Region Skåne. MS received research funding from Hansa Biopharma, Ingrid Asp Foundation, Inga-Britt & Arne Lundbergs Research Foundation, Swedish Renal Foundation, Region Skåne.

*Give information separately for cases and controls in case-control studies and, if applicable, for exposed and unexposed groups in cohort and cross-sectional studies.

Note: An Explanation and Elaboration article discusses each checklist item and gives methodological background and published examples of transparent reporting. The STROBE checklist is best used in conjunction with this article (freely available on the Web sites of PLoS Medicine at http://www.plosmedicine.org/, Annals of Internal Medicine at http://www.annals.org/, and Epidemiology at http://www.epidem.com/). Information on the STROBE Initiative is available at www.strobe-statement.org.
